# Efficient mRNA-Based Genetic Engineering of Human NK Cells with High-Affinity CD16 and CCR7 Augments Rituximab-Induced ADCC against Lymphoma and Targets NK Cell Migration toward the Lymph Node-Associated Chemokine CCL19

**DOI:** 10.3389/fimmu.2016.00105

**Published:** 2016-03-22

**Authors:** Mattias Carlsten, Emily Levy, Amrita Karambelkar, Linhong Li, Robert Reger, Maria Berg, Madhusudan V. Peshwa, Richard W. Childs

**Affiliations:** ^1^Laboratory of Transplantation Immunotherapy, Hematology Branch, National Heart, Lung, and Blood Institute, National Institutes of Health, Bethesda, MD, USA; ^2^MaxCyte Inc., Gaithersburg, MD, USA

**Keywords:** gene therapy, NK cells, NK cell immunotherapy, antibody-dependent cellular cytotoxicity, cellular migration

## Abstract

For more than a decade, investigators have pursued methods to genetically engineer natural killer (NK) cells for use in clinical therapy against cancer. Despite considerable advances in viral transduction of hematopoietic stem cells and T cells, transduction efficiencies for NK cells have remained disappointingly low. Here, we show that NK cells can be genetically reprogramed efficiently using a cGMP-compliant mRNA electroporation method that induces rapid and reproducible transgene expression in nearly all transfected cells, without negatively influencing their viability, phenotype, and cytotoxic function. To study its potential therapeutic application, we used this approach to improve key aspects involved in efficient lymphoma targeting by adoptively infused *ex vivo*-expanded NK cells. Electroporation of NK cells with mRNA coding for the chemokine receptor CCR7 significantly promoted migration toward the lymph node-associated chemokine CCL19. Further, introduction of mRNA coding for the high-affinity antibody-binding receptor CD16 (CD16-158V) substantially augmented NK cell cytotoxicity against rituximab-coated lymphoma cells. Based on these data, we conclude that this approach can be utilized to genetically modify multiple modalities of NK cells in a highly efficient manner with the potential to improve multiple facets of their *in vivo* tumor targeting, thus, opening a new arena for the development of more efficacious adoptive NK cell-based cancer immunotherapies.

## Introduction

Natural killer (NK) cells are cytotoxic immune cells that play an important role in the defense against cancer. They have also been shown to induce antitumor responses in settings of hematopoietic stem cell transplantation and in pilot clinical trials utilizing adoptive NK cell transfer (1, 2). Several methods to expand clinical-grade NK cells have recently been developed that allow for multiple injections of a large number of highly cytotoxic NK cells. Preliminary data from our ongoing phase I clinical trial have established that up to 2.5 × 10^8^ autologous *ex vivo*-expanded NK cells per kilogram can be safely infused into cancer patients, with tumor regression observed in some patients (3).

Genetic manipulation of NK cells to improve their persistence, tumor-targeting capacity, and the ability to home to disease sites *in vivo* may further enhance the efficacy of NK cell-based cancer immunotherapy (4). However, genetic manipulation of NK cells has historically proven to be challenging (5). In contrast to T cells, viral transduction of NK cells is less efficient and may compromise cell viability as summarized in Carlsten and Childs (5). Due to the use of viral vectors, this approach also comes with regulatory issues, high costs, and the need for specialized high-level biosafety laboratory platforms when taken to a clinical setting. Moreover, the predicted relatively short persistence of adoptively infused NK cells compared to T cells implies that stable transgene expression may not be equally necessary for this cell type. Therefore, we investigated mRNA electroporation as an alternative method to genetically modify NK cells for clinical use. This approach can genetically modify cells without using viral vectors, precluding the need for high-level biosafety laboratories. Although preclinical studies have shown that mRNA electroporation can be used to genetically modify NK cells (2, 6), a detailed characterization describing how electroporation affects NK cells *per se* and how this approach can be used to modify multiple modalities on one NK cell, such as tumor tissue homing and ability to target antibody-coated tumor cells, to further improve NK cell-based cancer immunotherapy has not yet been reported.

Here, we present detailed data characterizing the transgene expression, viability, proliferative capacity, phenotype, and cytotoxic function of *ex vivo*-expanded human NK cells following mRNA electroporation using a cGMP-compliant platform. Further, we demonstrate that this approach can be used to modify NK cells to improve their homing capacity to chemokines expressed in malignant lymphoid tissues and augment their ability to mediate antibody-dependent cellular cytotoxicity (ADCC). Collectively, our data demonstrate that mRNA electroporation is an efficient method to genetically modify NK cells, with the potential to reprogram multiple NK cell properties that boost their antitumor function without incurring any major negative effects on this cell population.

## Materials and Methods

### Cell Lines and Reagents

The K562, MM.1S, and CD20^+^ 721.221 cell lines were obtained from ATCC and propagated in RPMI 1640 supplemented with 10% heat-inactivated FBS (Sigma-Aldrich) and 2 mM glutamine (Life Technologies). The following reagents were used: anti-CD56 (NCAM-1), anti-CD3 (UCHT1), anti-CD16 (3G8), anti-NKG2D (1D11), anti-TRAIL (RIK-2), anti-NKp30 (p30-15), anti-NKp46 (29A1.4), IgG1 (MOPC21), and Annexin V and 7-AAD from Becton Dickinson (BD); anti-KIR2DL1/DS1 (EB6), anti-KIR2DL2/3/DS2 (GL183), and anti-NKG2A (Z199) from Beckman Coulter; the anti-CD107a (H4A3), anti-KIR3DL1 (Dx9), anti-CD57 (HCD57), anti-2B4 (C1.7), anti-CD34 (581), anti-CCR7 (G043H7), IgG2a (MOPC-173), and BV650-streptavidin from Biolegend; the anti-Lir-1 (HP-F1) from eBioscience; the anti-NKG2C (134591) from R&D Systems; LIVE/DEAD viability marker from Life Technologies; biotinylated anti-KIR3DL2 (Dx31) from UCSF; rituximab (Rituxan) from Genentech; and off-the-shelf eGFP mRNA and custom-made CD34, CD16, and CCR7 mRNAs from TriLink Biotechnology.

### NK Cell Expansion

Natural killer cells expanded *ex vivo* for 11–15 days were isolated from healthy donor PBMC using the NK cell isolation kit from Miltenyi and combined in G-Rex flasks (Wilson Wolf Manufacturing) with irradiated EBV–SMI–LCL cells at a ratio of 1:10 in NK cell media [X-VIVO 20 (Lonza) supplemented with 10% heat-inactivated human AB plasma (Sigma-Aldrich) and 500 IU/ml of recombinant human IL-2 (Roche)] (3). The cells were cultured at 37°C and 6.5% CO_2_. Half media was replaced with fresh NK cell media 5 days into the expansion. Thereafter, NK cells were counted and adjusted to 0.5–1 × 10^6^ cells/ml every 48 h, from day 7 until utilized in experiments.

### Electroporation of NK Cells

Natural killer cells were electroporated using the MaxCyte GT^®^ Transfection System. In brief, cells were first collected and washed in electroporation buffer (HyClone). They were then mixed with mRNA in a total volume of 100 μl and transferred to an OC-100 cuvette. Electroporation was conducted using an optimized program for NK cells. The instrument settings for optimized NK cell transfection are proprietary to MaxCyte, Inc. Cells were then transferred to one well of a 48-well plate and incubated at 37°C and 6.5% CO_2_ for 20 min before being resuspended in NK cell media and transferred to culture flasks.

### Cytotoxicity Assay

Natural killer cells were cocultured at a ratio of 1:1 with either ^51^Cr-labeled K562 cells or MM.1S cells in a final volume of 200 μl in 96-well plates at 37°C and 5% CO_2_. After 4 h, supernatant was harvested onto a Luma plate. Counts were measured using a Perkin Elmer 1450 Microbeta Counter and specific target lysis was calculated using the following formula: [(NK cell-induced ^51^Cr release − spontaneous ^51^Cr release)/(maximum ^51^Cr release − spontaneous ^51^Cr release) × 100].

### NK Cell Migration Assay

Migration assays were performed using 24-well plates with Corning Transwell^®^ inserts. Six hundred microliters of serum-free X-VIVO 20 containing various concentrations of recombinant human CCL19 (Biolegend) was added to the bottom chambers, and 5 × 10^4^ NK cells in 100 μl of serum-free X-VIVO 20 media without CCL19 was added to the top chambers. The plate was incubated for 2 h at 37°C in 5% CO_2_ before transwell membranes were removed and cells in the bottom chamber were harvested. The amount of migrated cells was quantified on a Wallac 1420 Microplate Reader (Perkin Elmer) using the CyQUANT kit (Life Technologies). Cells plated straight to the bottom chamber were used as maximum control, and the proportion of migrated cells was calculated as a percent of total cells initially added to each well.

### NK Cell Degranulation Assay

Natural killer cells were cocultured with 721.221 cells at a ratio of 1:1 in 96-well plates at 37°C and 5% CO_2_ with or without rituximab. After 1 h, cells were stained with cell surface mAbs and a viability marker for 15 min on ice, followed by washes and fixation in 1% paraformaldehyde (MP Biomedicals) in PBS. Cells were acquired on a BD LSR II Fortessa.

### Data and Statistical Analysis

Flow cytometry data were analyzed using the FlowJo software (Treestar, Inc.). Graphs and statistical analyses were performed with GraphPad PRISM (GraphPad Software, Inc.). *p < 0.05 and **p < 0.01.

## Results

### High Transfection Efficacy following mRNA Electroporation of NK Cells without Compromising Their Viability and Function

**Figure 1:**
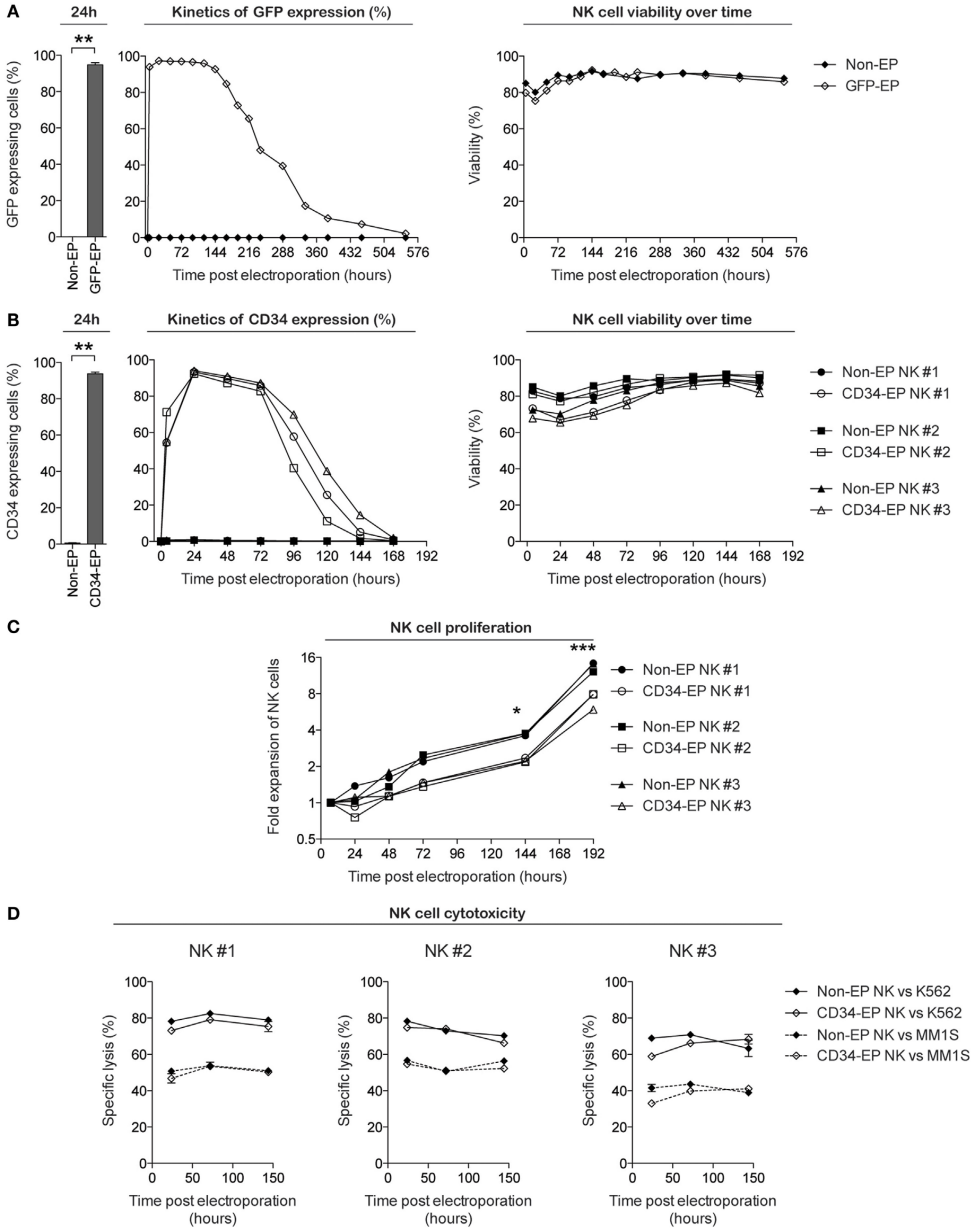
**Characterization of clinical-grade *ex vivo*-expanded human NK cells electroporated with GFP and CD34 mRNA**. *Ex vivo*-expanded NK cells were electroporated with mRNA coding for GFP or CD34 using the MaxCyte GT instrument. Transgene expression, viability, and proliferative and cytotoxic capacity were measured over time using flow cytometry. **(A)** GFP expression and viability (determined by Annexin V and 7-AAD from Becton Dickinson) of *ex vivo*-expanded NK cells from healthy donors following electroporation with 0.25 μg of eGFP mRNA (TriLink Biotechnology) per million NK cells. **(B)** CD34 expression (determined by anti-CD34 antibody staining) and viability of NK cells expanded from three healthy donors following electroporation with 1 μg of CD34 mRNA (TriLink Biotechnology) per million NK cells. **(C)** Fold expansion of NK cells *ex vivo* following CD34 mRNA electroporation compared to non-electroporated NK cells. **(D)** Specific lysis of K562 cells and the multiple myeloma cell line MM.1S by CD34 mRNA-electroporated and non-electroporated NK cells. Non-EP, non-electroporated; GFP-EP, eGFP mRNA electroporated; CD34-EP, CD34 mRNA electroporated. A Wilcoxon ranked sum *t*-test was used in **(A,B)**, whereas a paired *t*-test was used in **(C)**.

We first set out to evaluate the transfection efficacy, viability, proliferation, and function of *ex vivo*-expanded NK cells following mRNA electroporation. Using the scalable cGMP-compliant MaxCyte GT transfection system and expanded NK cells utilizing methods from our ongoing phase I clinical trial, we observed highly reproducible rapid GFP protein expression in nearly 100% of eGFP mRNA-electroporated NK cells (Figure 1A). GFP expression was detectable in proliferating NK cells for up to 3 weeks, with expression in >95% of cells for the first 7–9 days after gene delivery and had no deleterious effects on cell viability. Since GFP represents an intracellular molecule with a long half-life, we also evaluated the capacity of this transfection technique to induce expression of selected molecules on the surface of NK cells. Electroporation of NK cells with mRNA coding for the cell surface marker CD34 led to surface expression for up to 7 days, with peak expression occurring approximately 24 h after electroporation (Figure 1B). As with GFP, no significant impact on viability was observed following electroporation. Although electroporated NK cells maintained their capacity to proliferate *in vitro*, there was a slight delay in the start of proliferation after electroporation compared to non-electroporated controls (Figure 1C). Importantly, NK cell cytotoxicity, as assessed by their ability to kill K562 cells and the multiple myeloma cell line MM.1S, remained high and was unaffected by electroporation compared to controls (Figure 1D).

### mRNA Electroporation of NK Cells Does Not Significantly Alter the Expression of NK Cell Surface Receptors

**Figure 2:**
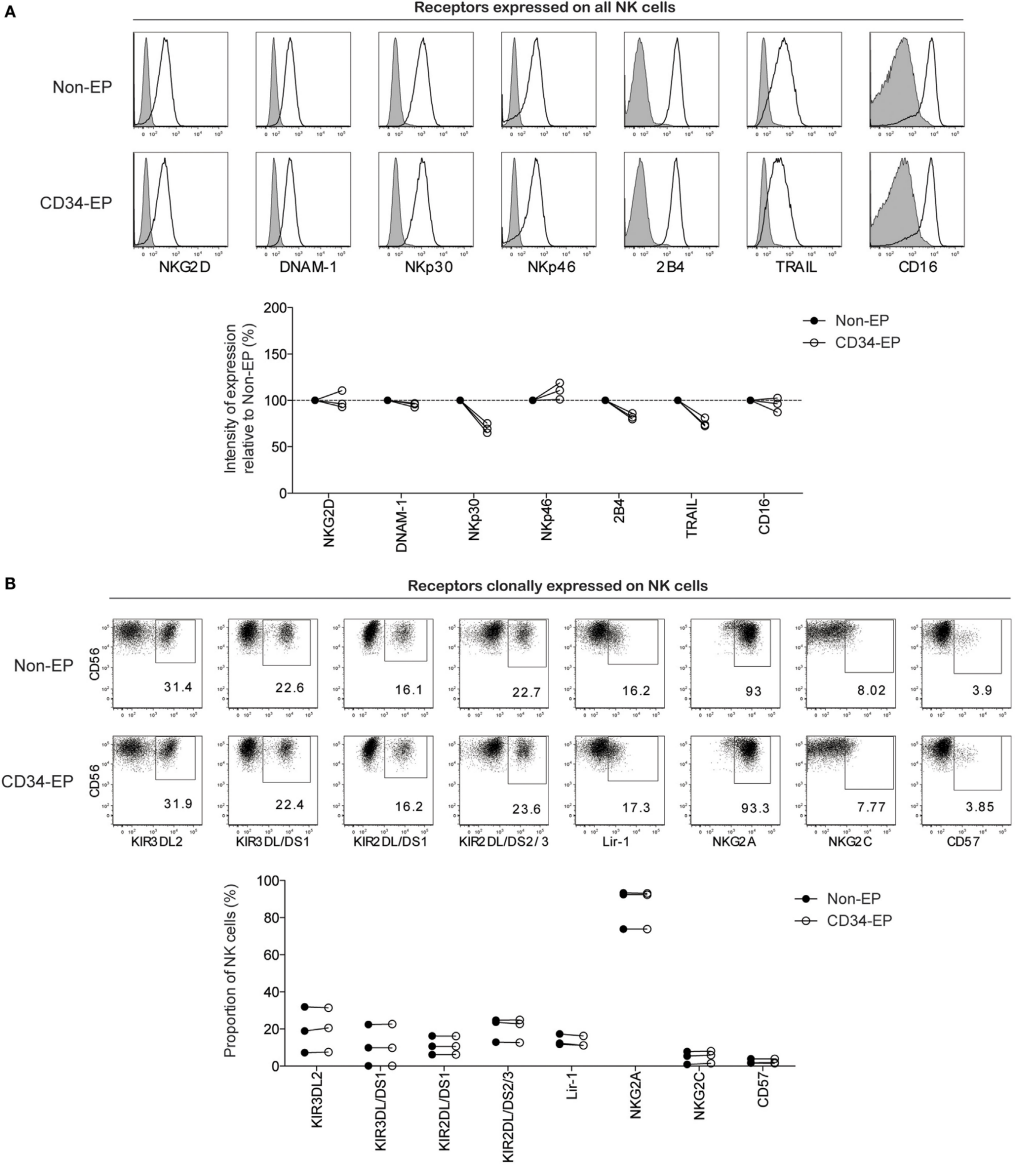
**Phenotypic characterization of clinical-grade *ex vivo*-expanded NK cells following mRNA electroporation**. Expression of activating and inhibitory NK cell receptors on *ex vivo*-expanded NK cells was assessed 24 h after electroporation with CD34 mRNA (1 μg/million NK cells) using the MaxCyte GT instrument. **(A)** Representative histograms for one NK cell donor and pooled data from three donors showing the relative expression intensity of selected NK cell receptors on electroporated compared to non-electroporated *ex vivo*-expanded NK cells. **(B)** Representative dot plots for one NK cell donor and pooled data from three donors showing expression of clonally expressed NK cell receptors on electroporated and non-electroporated NK cells from three healthy donors. Non-EP, non-electroporated; CD34-EP, CD34 mRNA electroporated.

As electroporation of NK cells potentially could lead to unintended phenotypic alternations that perturb NK cell activation and target specificity, we next performed high-resolution flow cytometry to evaluate the phenotype of CD34 mRNA-electroporated NK cells compared to controls. With the exception of slight decreases in NKp30, 2B4, and TRAIL, there was no significant alteration in the surface expression of any of the 15 activating and inhibitory NK cell receptors in CD34 mRNA-electroporated NK cells compared to controls 24 h after electroporation (Figure 2). Daily flow cytometry analysis showed no additional phenotypic changes occurred during the first 6 days following electroporation with CD34 mRNA (data not shown).

### Reprograming of NK Cells with the Chemokine Receptor CCR7 Improves Their Ability to Migrate Toward the CCR7 Ligand CCL19

**Figure 3:**
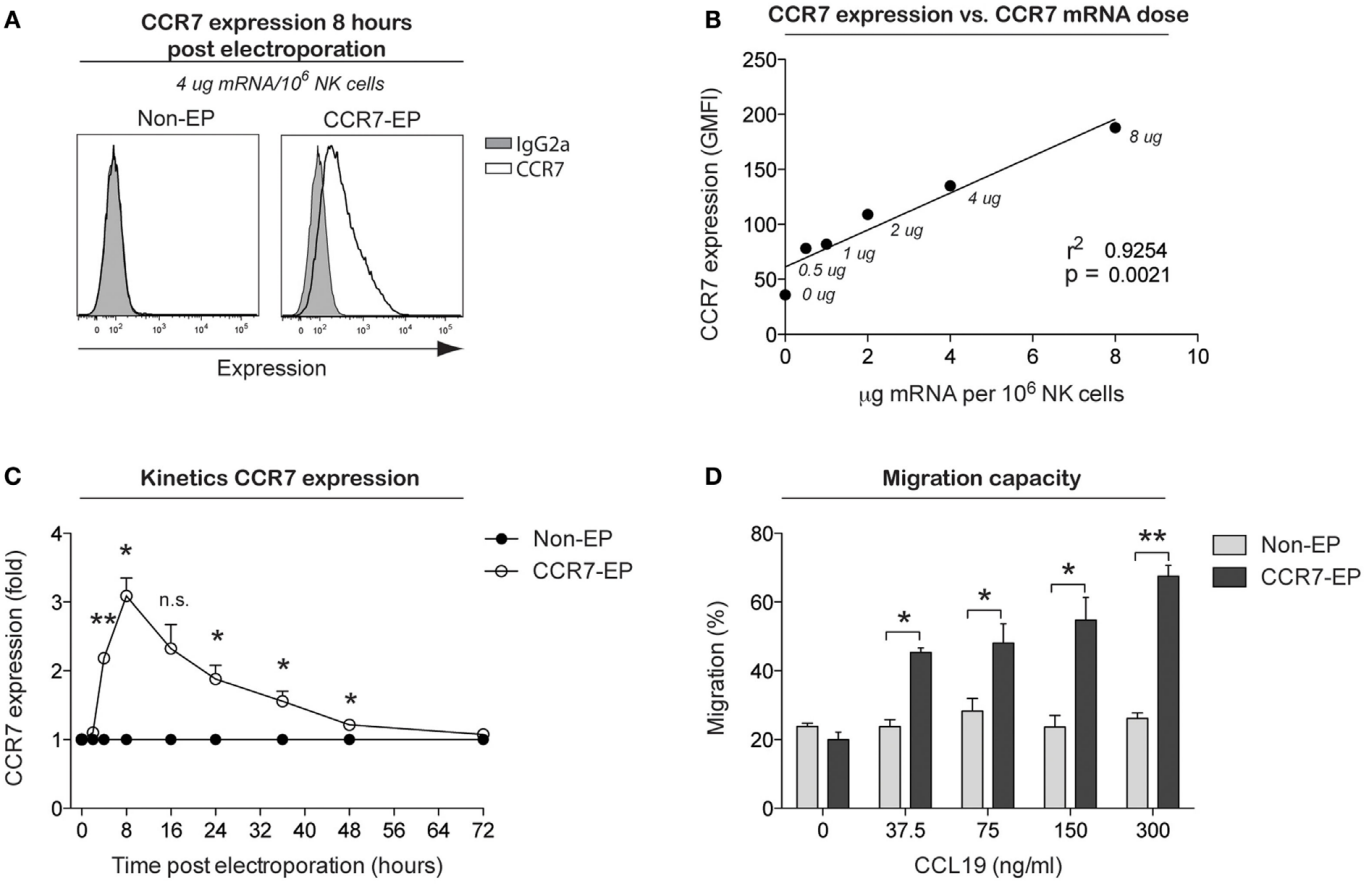
**CCR7 expression and migration capacity of clinical-grade *ex vivo*-expanded NK cells electroporated with CCR7 mRNA**. *Ex vivo*-expanded NK cells were electroporated with mRNA coding for the homing receptor CCR7 using the MaxCyte GT instrument. **(A)** Representative example of CCR7 expression on NK cells 8 h after electroporation with CCR7 mRNA (CCR7-EP) compared to non-electroporated (non-EP) NK cells. **(B)** Correlation between CCR7 expression and CCR7 mRNA dose (line; linear regression). **(C)** Kinetics of CCR7 expression on NK cells following electroporation with 4 μg/million NK cells. Error bars, SEM. **(D)** Transwell migration of non-EP and CCR7-EP NK cells against a gradient of CCL19 (a ligand for CCR7). Error bars, SEM. A paired *t*-test was used in **(C,D)**.

The use of mRNA electroporation to genetically engineer NK cells to improve their homing capacity has not previously been studied. Therefore, we next electroporated NK cells with mRNA coding for the chemokine receptor CCR7, which is known to direct cellular migration to secondary lymphoid tissues, including lymph nodes where hematological malignancies such as lymphoma reside. The CCR7 receptor is normally expressed by only a small subset of primary NK cells (primarily the CD56^bright^ NK cell subset), with expression completely disappearing following *ex vivo* expansion. By electroporating expanded NK cells with increasing concentrations of CCR7 mRNA, a correlation between mRNA dose and CCR7 cell surface expression was established (Figures 3A,B). Using 4-μg CCR7 mRNA per million NK cells, cell surface expression of CCR7 was sustained for up to 48 h with peak expression measured 8 h following electroporation (Figure 3C). Except for increased TRAIL expression, no changes in NK cell receptor expression were observed on CCR7 mRNA-electroporated NK cells compared to their non-electroporated counterparts (Figure S1 in Supplementary Material). Moreover, as with CD34 mRNA-electroporated NK cells, NK cells electroporated with CCR7 mRNA maintained potent cytotoxicity function against tumor cells (Figure S2 in Supplementary Material). Importantly, CCR7 mRNA-electroporated NK cells showed marked dose dependent *in vitro* migration capacity toward the CCL19 chemokine (Figure 3D), in contrast to non-electroporated NK cells, which lacked CCL19-specific migration. As reported for other CCR7-expressing lymphocytes, exposure of CCR7 mRNA-electroporated NK cells to CCL19 led to a dose-dependent reduction of cell surface CCR7 expression (Figure S3 in Supplementary Material) (7), indicating that the transfected receptor behaved similar to wild-type CCR7.

### Augmented NK Cell ADCC by Introduction of the High-Affinity CD16-158V Receptor

**Figure 4:**
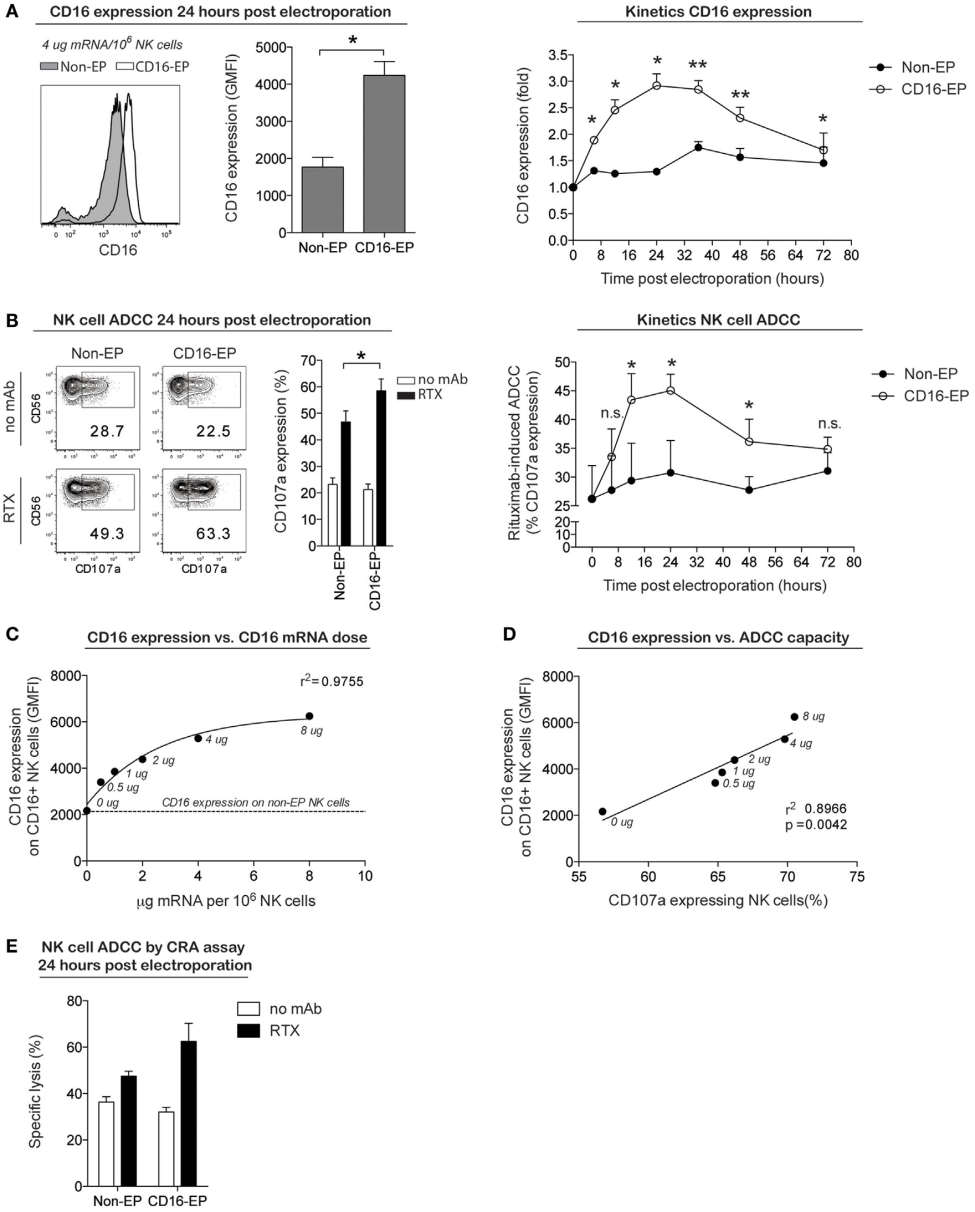
**CD16 expression and ADCC capacity of clinical-grade *ex vivo*-expanded NK cells electroporated with CD16-158V mRNA**. *Ex vivo*-expanded NK cells were electroporated with mRNA coding for the high-affinity Fc receptor CD16-158V using the MaxCyte GT instrument. **(A)** Representative example and average cell surface expression of CD16 on NK cells 24 h after electroporation with CD16 mRNA (CD16-EP) compared to non-electroporated (non-EP) NK cells (*n* = 7) as well as kinetics of CD16 surface expression following electroporation (*n* = 3) Error bars, SEM. **(B)** NK cell degranulation (measured by CD107a expression) by CD16-158V mRNA-electroporated NK cells compared to non-EP NK cells following coculture with CD20^+^ 721.221 EBV–LCL cells in the absence (no mAb, no monoclonal antibody) and presence of rituximab (RTX) 24 h after electroporation (*n* = 7) as well as kinetics of NK cell ADCC following electroporation (*n* = 3). Error bars, SEM. **(C)** Correlation between CD16 expression and CD16-158V mRNA dose [line; variable slope log (agonist) vs. response regression]. **(D)** Correlation between NK cell CD16 expression and ADCC capacity against rituximab-treated 721.221 EBV–LCL (line; linear regression). **(E)** Specific lysis of CD20^+^ 721.221 EBV–LCL cells by CD16-158V mRNA-electroporated NK cells compared to non-EP NK cells [as measured in a ^51^Cr-release assay (CRA) 24 h after electroporation] in the absence (no mAb, no monoclonal antibody) and presence of rituximab (RTX) at a 0.5:1 ratio of NK cells to EBV–LCL targets. Error bars, SD. Wilcoxon ranked sum *t*-tests were used in the bar graphs in **(A,B)**, whereas paired *t*-tests were used for statistics in the graphs showing kinetics of CD16 expression and NK cell ADCC.

To establish whether this cGMP-complaint transfection technique could also be used to improve NK cell cytotoxicity, NK cells obtained from CD16-158F/F donors were transfected with mRNA coding for the high-affinity Fc receptor CD16-158V in an effort to augment their capacity to induce ADCC against rituximab-coated CD20^+^ B cell lymphoma cells. As shown in Figure 4A, 24 h following the electroporation of NK cells with CD16-158V mRNA, cell surface expression of CD16 increased significantly compared to non-electroporated controls. As for CD34 and CCR7 mRNA-electroporated NK cells, no major changes in NK cell receptor expression was observed on NK cells electroporated with CD16-158V mRNA (Figure S1 in Supplementary Material). Kinetic studies showed CD16 expression on electroporated cells remained at higher levels than controls for up to 72 h following electroporation. In line with these phenotypic changes, CD16-158V mRNA-electroporated NK cells acquired an enhanced ability to mediate ADCC when cocultured with rituximab-coated EBV-transformed B-cell lymphoma cells compared to controls (Figures 4B,E). This enhanced killing effect against rituximab-treated tumor cells persisted for up to 3 days following electroporation (Figure 4B). By conducting experiments where NK cells were loaded with different concentrations of the CD16-158V mRNA, we observed that both CD16 expression and ADCC were CD16-158V mRNA dose dependent (Figures 4C,D). Importantly, electroporation of NK cells with CD16-158V mRNA did not affect their baseline (non-ADCC-mediated) cytotoxic function (Figure S2 in Supplementary Material). Taken altogether, these data establish mRNA electroporation as a rapid, efficient, and non-toxic method to genetically modify *ex vivo*-expanded NK cells to improve their tumor homing capacity and antitumor cytotoxic function.

## Discussion

Here, we show that mRNA electroporation is a highly efficient method to genetically reprogram multiple NK cell modalities without incurring any deleterious effects on their viability, phenotype, and function. The data presented in this report provide a foundation for strategies that incorporate mRNA electroporation into clinical trials utilizing adoptive NK cell immunotherapy. The electroporation system used in these experiments on NK cells is ideally suited for use in the clinic, having the capacity to transfect extremely large numbers of cells (up to 2 × 10^10^ cells) in only 20 min under GMP conditions. Finally, the recent use of this system in clinical trials of dendritic cell vaccine and T cell-based cancer therapies further highlights the ready applicability of mRNA electroporation into NK cell-based immunotherapy trials (8, 9).

Compared to viral transduction, mRNA electroporation results in relatively transient protein expression. In this context, variability in the half-life of proteins as well as variability in membrane turnover and receptor internalization are the factors that regulate surface expression time for specific transfected proteins. Modifications of the mRNA, such as capping, poly(A) elongation, and use of pseudouridine instead of uridine nucleotides, may all be used to prolong the time of transgene expression. However, it is important to consider that adoptively infused NK cells can mediate rapid antitumor responses while persisting for relatively short periods compared to adoptively transferred T cells. Therefore, stable transgene expression in these cells may not be necessary. Indeed, the short-lived and non-integrating nature of mRNA may be advantageous in situations where new transgenes are being tested for the first time in patients, as this approach potentially minimizes the risk of uncontrollable adverse events, which have been observed to occur following the infusion of stably transduced T cells (10, 11). The development of methods that expand large numbers of NK cells *ex vivo* from the blood of cancer patients now provides a clinical scenario, where repeated infusions of genetically modified NK cells can be used to improve the efficacy of adoptive NK cell transfer while reducing risks related to unexpected side effects (3).

The availability of the GMP-compliant platform described herein offers a method to explore a wide range of genetic manipulations to promote NK cell-based cancer immunotherapy. Genetic manipulation of NK cells to target tumors through expression of chimeric antigen receptors (CARs) represents one of the more attractive clinical uses of this approach (7, 12). As previously demonstrated (2, 6), NK cells electroporated with mRNA coding for an anti-CD19 CAR acquired augmented cytotoxicity against B cell malignancies both *in vitro* and in a preclinical animal model. Our data suggest that mRNA electroporation could also be used to augment the ability of adoptively transferred NK cells more globally by enhancing their ability to mediate ADCC in patients with cancer who are receiving treatment with monoclonal antibodies. In the clinic, it has been recognized that lymphoma patients homozygous for the CD16-158V polymorphism, encoding for a high-affinity IgG antibody-binding receptor, have substantially higher response rates following treatment with the anti-CD20 antibody rituximab compared to those who carry the low-affinity CD16-158F polymorphism (13–16). Preclinical data presented in this study suggest the infusion of *ex vivo*-expanded NK cells electroporated to express the CD16-158V receptor could enhance antibody-mediated antitumor responses through ADCC. This approach is appealing not only because a minority of patients are homozygous for the CD16-158V polymorphism but also since infusions of CD16-158V electroporated NK cells could be used to improve the efficacy of virtually any malignancy for which there is an FDA approved IgG1 antibody.

Another group of receptors that could be modified to improve outcomes following NK cell-based cancer immunotherapy are homing receptors. Based on preclinical mouse data showing improved lymph node homing of CCR7-overexpressing NK cells (17) along with data presented in this study, electroporation of NK cells with mRNA coding for CCR7 is a candidate strategy to study in patients with lymphoma as well as in patients who have tumors metastasized to lymphoid tissues. If combined with CD16 mRNA electroporation, this approach could theoretically be used to improve the ability of adoptively transferred NK cells to both home to tumor cells residing in lymphoid tissues and kill these lymphoid residing tumor cells *via* augmented NK cell ADCC following monoclonal antibody treatment.

Other applications for this technology include reprograming of NK cells by electroporation of mRNA coding for transcription factors or by combining it with the CRISPR/Cas9 technology, whereby guiding RNAs together with mRNA coding for the endonuclease Cas9 are introduced to silence genes by inducing DNA double-strand breaks. The latter can be used to permanently deplete genes coding for inhibitory receptors expressed on NK cells, such as programed cell death-1 (PD-1), thereby increasing their tumor-targeting capacity.

In conclusion, these studies establish mRNA electroporation to be a highly efficient method to express transgenes in *ex vivo*-expanded clinical-grade NK cells without incurring any negative effects on their phenotype and antitumor function. The availability of this platform offers a novel, rapid, and efficient strategy to genetically manipulate NK cells and opens up numerous new possibilities to advance the field of NK cell-based cancer immunotherapy. Future clinical trials studying the adoptive infusion of NK cells genetically reprogramed to more efficiently home to tumor-bearing tissues and thereby better recognize and kill the tumor cells will soon shed light on the potential of this approach in patients with cancer.

## Author Contributions

MC: design, experiments, data analysis, figures, and manuscript writing; EL, AK, LL, and RR: experiments and manuscript review; MB and MP: manuscript review; and RC: design and manuscript writing.

## Conflict of Interest Statement

LL and MP are employees of MaxCyte, Inc. The remaining authors have no conflicts of interest to declare.
