# Supplementary material for: Efficient mRNA-Based Genetic Engineering of Human NK Cells with High-Affinity CD16 and CCR7 Augments Rituximab-Induced ADCC against Lymphoma and Targets NK Cell Migration toward the Lymph Node-Associated Chemokine CCL19
Source: Front Immunol. 2016 Mar 22;7:105. doi: 10.3389/fimmu.2016.00105 (PMC4801851; doi:10.3389/fimmu.2016.00105)
Supplement: Supplementary file 1 [file Presentation_1.PDF]

Supplemental Figure 1. Carlsten et al.

A

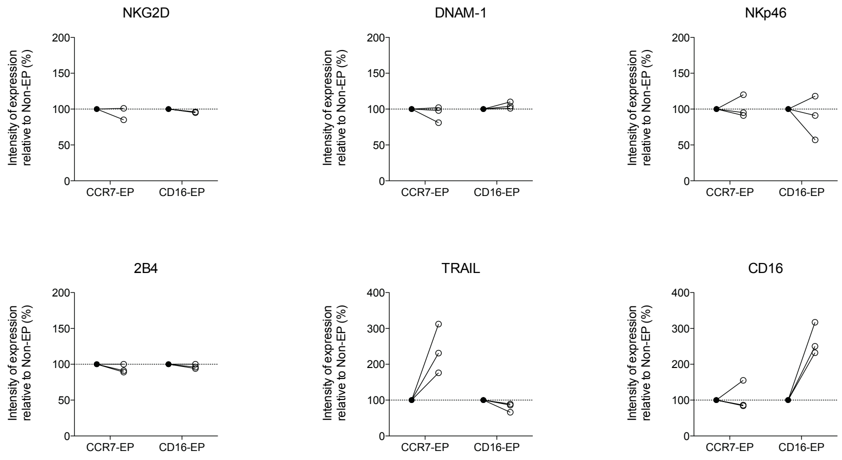

B

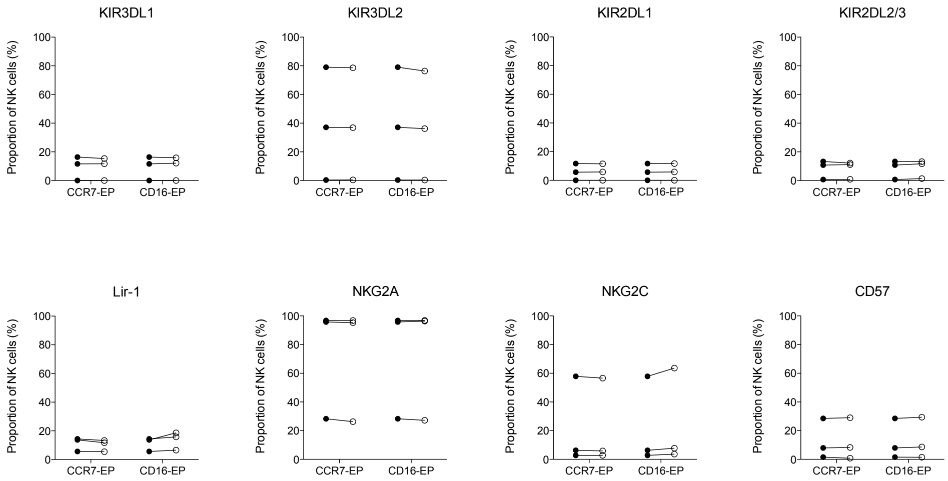

Supplemental Figure 2. Carlsten et al.

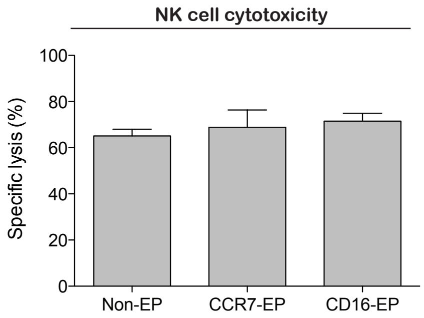

Supplemental Figure 3. Carlsten et al.

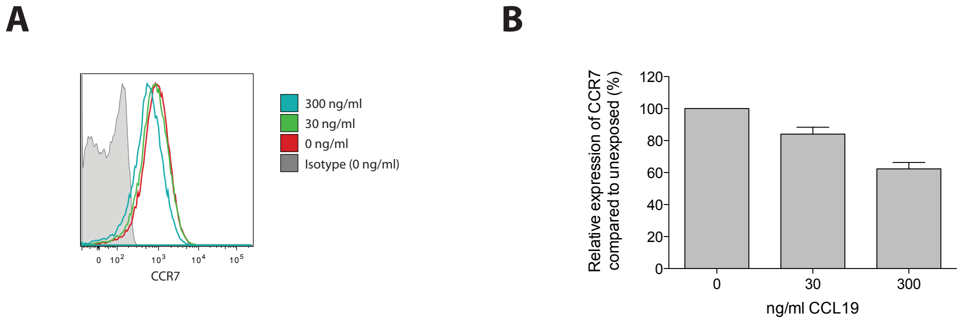

Supplemental Figure Legends

Supplemental Figure 1. Phenotypic characterization of clinical-grade *ex vivo* expanded NK cells following electroporation with mRNA coding for CCR7 and CD16. Expression of activating and inhibitory NK cell receptors on *ex vivo* expanded NK cells was assessed 24 hours after electroporation with CCR7 and CD16 mRNA (2 ug per million NK cells) using the MaxCyte GT instrument. (A), Data from three donors showing the relative expression intensity of selected NK cell receptors on electroporated (open circles) compared non-electroporated (filled circles) *ex vivo* expanded NK cells. (B), Data from three donors showing expression of clonally expressed NK cell receptors on electroporated (open circles) and non-electroporated (filled circles) NK cells from three healthy donors. *Non-EP, non-electroporated. CCR7-EP, CCR7 mRNA electroporated. CD16-EP, CD16 mRNA electroporated.*

Supplemental Figure 2. Functional characterization of clinical-grade *ex vivo* expanded human NK cells electroporated with CCR7 and CD16 mRNA. *Ex vivo* expanded NK cells were electroporated with mRNA coding for CCR7 or CD16 using the MaxCyte GT instrument. Their cytotoxic capacity against K562 cells was measured at Effector-to-Target ratio 1:1 in ^{51}Cr release assays and compared to that of non-electroporated NK cells. *Non-EP, non-electroporated. CCR7-EP, CCR7 mRNA electroporated. CD16-EP, CD16 mRNA electroporated. Bars, mean of three donors. Error bars, standard error of the mean.*

Supplemental Figure 3. CCR7 expression on clinical-grade *ex vivo* expanded human NK cells electroporated with CCR7 mRNA following exposure to the CCR7 ligand CCL19. *Ex vivo* expanded NK cells were electroporated with mRNA

coding for CCR7 using the MaxCyte GT instrument. CCR7 cell surface expression was analyzed 10 hours post electroporation following cellular exposure to different concentrations of the CCR7 ligand CCL19 during the last 2 hours. (A), Histogram showing a representative example of the CCR7 expression on CCR7 mRNA electroporated NK cells from donor following exposure to different concentrations of CCL19 for 2 hours. (B), Data from three donors showing relative changes in the expression of CCR7 on CCR7 mRNA electroporated NK cells following exposure to different concentrations of CCL19 for 2 hours. *Bars, mean of three donors. Error bars, standard error of the mean.*
